# Hamilton Rating Scale for Anxiety: exploring validity with robust measures of classical theory parameters and a rating scale model in university students

**DOI:** 10.1192/bjo.2025.10055

**Published:** 2025-08-12

**Authors:** Md Dilshad Manzar, Faizan Z. Kashoo, Mohammed Salahuddin, Dejene Nureye, Habtamu Acho Addo, Seithikurippu R. Pandi-Perumal, Amir H. Pakpour, Ahmed S. Bahammam

**Affiliations:** Department of Primary Nursing Care, College of Nursing, Majmaah University, Majmaah, Saudi Arabia; Department of Physical Therapy and Health Rehabilitation, College of Applied Medical Sciences, Majmaah University, Majmaah, Saudi Arabia; Department of Pharmaceutical Sciences, School of Pharmacy, Notre Dame of Maryland University, Baltimore, Maryland, USA; School of Pharmacy, College of Medicine and Health Sciences, Mizan-Tepi University, Mizan-Aman, Ethiopia; Research Unit of Neuroinflammatory and Cardiovascular Pharmacology, Faculty of Science, University of Dschang, Dschang, Cameroon; Centre for Research and Development, Chandigarh University, Mohali, Punjab, India; Division of Research and Development, Lovely Professional University, Phagwara, Punjab, India; Department of Nursing, School of Health and Welfare, Jönköping University, Jönköping, Sweden; University Sleep Disorders Center, Department of Medicine, College of Medicine, King Saud University, Riyadh, Saudi Arabia; National Plan for Science and Technology, College of Medicine, King Saud University, Riyadh, Saudi Arabia

**Keywords:** Reliability, validity, item response theory, psychometrics, Ethiopia

## Abstract

**Background:**

No research has assessed Hamilton Rating Scale for Anxiety (HRSA) psychometric properties in Ethiopian university students, using item response theory (IRT) and classical theory.

**Aims:**

This study aimed to assess psychometric properties of the English HRSA in Ethiopian students, using IRT and classical theory.

**Method:**

University students (*N* = 370, age 21.44 ± 2.30 years) in Ethiopia participated in a cross-sectional study. Participants completed a self-reported measure of anxiety, a sociodemographics tool and interviewer-administered HRSA.

**Results:**

Confirmatory factor analysis (CFA) favoured a one-factor structure because fit indices for the one-factor model; and two distinct two-factor models were similar, but high interfactor correlations violated discriminant validity criteria in two-factor models. This one-factor structure showed structural invariance as evidenced by multi-group CFA across gender groups. No ceiling/floor effects were seen for the HRSA total scores. Infit and outfit mean square values for all the items were within the acceptable range (0.6–1.4). Four threshold estimates (*τ*i1, *τ*i2, *τ*i3 and *τ*i4) for each item were ordered as expected. Differential item functions showed item-level measurement invariance for all the 14 HRSA items across gender for both uniform and non-uniform estimates. McDonald’s *ω* and Cronbach’s *α* for the HRSA tool were both 0.88. The convergent validity of the interviewer-administered HRSA with self-reported anxiety subscale of the 21-item Depression, Anxiety and Stress Scale was weak to moderate.

**Conclusions:**

The findings favour the validity of a one-factor structure of the HRSA with adequate item properties (classical and rating scale model), convergent validity, reliability and measurement invariance (structural and item level) across gender groups in Ethiopian university students.

Anxiety disorders are a common mental health condition that can significantly affect an individual’s quality of life. The World Health Organization estimates that 301 million people have anxiety disorders, with approximately 20% of them being children and adolescents.^1^ Patients diagnosed with anxiety disorder are three to five times more likely to visit the doctor and six times more likely to be admitted to hospital for other psychiatric problems compared with those who were not diagnosed with an anxiety disorder.^2,3^ Excessive fear, worry and behavioural abnormalities are some of the hallmark traits of anxiety disorders^1^. Anxiety disorders can also significantly impair an individual’s ability to perform daily activities, maintain employment and engage in social interactions.^4^ Intriguingly, the COVID-19 pandemic has exacerbated stress and anxiety levels by 25%, especially among youth and women.^1^ A recent systematic review reported a global prevalence of anxiety of 27–30%.^5,6^ Furthermore, in 2020, anxiety disorders caused 44.5 million disability-adjusted life-years (DALYs) globally.^7^

University students at colleges and universities are the most vulnerable population who might require mental health intervention services.^8^ The transitional phase of emerging adulthood, which typically occurs during the university years, can indeed be a period filled with various life events and significant stressors ranging from starting college to leaving home and moving to a new city and making new friends to taking finals and looking for a job.^9^ However, many university students do not seek mental health treatments because of concerns regarding confidentiality, time and cost constraints, unpleasant experiences with professional help and greater reliance on family and friends for support.^10,11^ Failure to timely address the mental health needs of university students is associated with poor academic performance, behavioural issues, dropping out, substance misuse, school violence and suicide.^9^ Furthermore, university students from low-income countries may go undetected for mental health illnesses because of the lack of a sophisticated infrastructure to screen for such disorders. In this regard, interviewer-administered anxiety screening methods may be beneficial for preliminary screening for such illnesses.

Anxiety disorders remain underdiagnosed, with only a minority detected in primary care services, presumably because of the underutilisation of diagnostic questionnaires in routine practice, as well as the cost and time involved in consultations.^12^ Therefore, simple, quick questionnaires are needed for routine practice. The Hamilton Rating Scale for Anxiety (HRSA) is a self-reported interviewer-administered questionnaire comprised of 14 questions that assess anxiety.^13^ A thorough psychometric validation of the HRSA in the Ethiopian population may help in establishing an evidence-based application as an initial screening tool for anxiety in this resource-limited setting. Such a prospective clinical application may help in the expansion of cost-effective screening for mental health issues of vulnerable groups. The HRSA is a widely used assessment tool for measuring anxiety symptoms. Unlike other tools such as the Generalised Anxiety Disorder Scale (GAD-7) and the Depression, Anxiety and Stress Scale (DASS-21), the HRSA can detect physiological and psychological symptoms.^14^ The HRSA questionnaire is valid in various primary healthcare settings.^14–16^ Although the HRSA is a widely used measure for screening anxiety symptoms, there is a relative scarcity of research using item response theory (IRT) framework and classical theory parameters.^17^ IRT modelling has several distinct advantages over classical test theory, such as the modelling of item-latent variable relationships (θ) with nonlinear functions, item parameters in the latent attribute metric and conditional precision parameters.^18–22^ Through IRT analysis, we could evaluate the psychometric properties of each item, examine their discrimination power, assess the overall scale functioning and explore potential improvements to enhance measurement precision in this context of use.^20–22^ However, only few studies have investigated the psychometric validity of the HRSA employing structural invariance tests, differential item functioning, Wright map and item characteristic curves (ICCs).

Therefore, in this study, the psychometric validity of the HRSA was investigated with an elaborate list of measures from both IRT and robust classical theory parameters, in a sample of Ethiopian university students.

## Method

### Participants and study design

We conducted an observational study with cross-sectional data collection and random sampling. A sample of 500 students from the Mizan campus of Health Science at Mizan-Tepi University (MTU) was earmarked for participation. Random samples were drawn from class sections comprising pharmacy, midwifery and nursing students. All selected students were extended invitations to participate in the study. The inclusion criteria were registration in MTU courses at the time of the study. Students under 18 years were not included as their participation may involve obtaining the consent of their parents and guardians, most of whom are usually located in remote areas. The findings from a sample (*N* = 370, mean age 21.44 ± 2.30 years) who completed this study are presented. Participants were given a summary of the research plan in simple language. A researcher’s contact detail was shared with participants to contact if they had any doubt or needed any more information. They were informed that all personal data would be kept confidential and that participation was voluntary. There were no rewards and no risks to participants’ health. Participants were free to withdraw at any time without any liabilities, and informed written consent was collected from the participants. The Ethical Institutional Committee, College of Medicine and Health Sciences, MTU, Ethiopia, approved the research plan (approval number DoP/0281/17). The authors assert that all procedures contributing to this work comply with the ethical standards of the relevant national and institutional committees on human experimentation and with the Helsinki Declaration of 1975, as revised in 2013.

Participating students completed the HRSA, DASS-21 and a sociodemographic information sheet in English. The HRSA was administered by researchers who were faculty members of the College of Medicine and Health Sciences, MTU. These faculty members were trained in the interpretation and assessment of interpreting and assessing the disease condition. The students filled in the DASS-21 and the sociodemographic information sheet. Participating students have adequate English proficiency as the mode of education in MTU.

### Instruments

#### The HRSA

The HRSA is a 14-item brief questionnaire to measure the severity of anxiety symptoms.^13^ All items are rated on a Likert scale of 0 (not present) to 5 (very severe). Item scores are added to get a total score (range: 0–56). Higher scores indicate higher severity of anxiety symptoms.^13^ The HRSA is widely used in both clinical and research settings. Additionally, the HRSA has been shown to have adequate validity in adolescents and the general population, mostly using classical theory parameters.^23,24^

#### Sociodemographic questionnaire

Participating students completed a brief structured questionnaire for sociodemographic information. In addition, information regarding age (years), ethnicity, gender, grade at the most recent examination, self-reported presence of chronic disease/conditions, failure to pass the previous examination and class attendance were collected.

#### The DASS-21

The DASS-21 is the shortened version of a questionnaire tool with 42 items, i.e. DASS-42. The DASS-21 was developed by Lovibond and Lovibond in 1995.^25^ All the items were scored on a scale of 0 (‘Did not apply to me at all’) to 3 (‘Applied to me very much or most of the time’). The DASS-21 has 21 items and three subscales, depression, anxiety and stress, with seven items each. Item scores were added to generate subscale scores (range: 0–21). Higher scores for the DASS-21 subscales indicate increasing severity of depression, anxiety and stress symptoms. The DASS-21 is valid in various settings and populations.^26^ In this study, the anxiety subscale of DASS-21 was used to determine the convergent validity of the HRSA.

### Data analysis

SPSS (version 23.0 for Windows), JASP (version 0.17.0.0 for Windows; JASP Team, Amsterdam, The Netherlands; https://jasp-stats.org) and JAMOVI (version 2.3.18 for Windows; The Jamovi Project, Sydney, Australia; https://www.jamovi.org) were used for the statistical analysis. Descriptive parameters were used to present participant characteristics and the HRSA item score distribution. The distribution of the HRSA item scores was deemed suitable for performing factor analysis based on Bartlett’s test of sphericity (*χ*^2^(91) = 2267.11, *P* < 0.001, Kaiser–Meyer–Olkin (KMO) test of sampling adequacy 0.90), and most of the inter-item correlation coefficients (75 out of 91) were 0.3 and above (Supplementary Table 1 available at https://doi.org/10.1192/bjo.2025.10055).^27,28^ As the HRSA item scores are ordinal variables, confirmatory factor analysis (CFA) was performed with diagonally weighted least squares (DWLS) with a pairwise deletion method of handling missing values, using JASP 0.17.0.0. Standardised estimates of factor loading with robust standard error were determined. CFA assessed the validity of the original one-factor as well as two distinct two-factor models that had been investigated in previous studies.^24,29^ As indicated by recent systematic reviews, we estimated multiple indices to unambiguously assess the fit of a model.^27,28^ The following fit indices were used: Bollen’s incremental fit index (IFI), parsimony normed fit index (PNFI), chi-squared (χ^2^) test, comparative fit index (CFI), goodness of fit index (GFI), root mean square error of approximation (RMSEA) and standardised root mean square residual (SRMR). A value of 0.9 or higher for the CFI, IFI and GFI, and a value of 0.08 or lesser for the SRMR, and RMSEA were taken to indicate a good fit. Moreover, a nonsignificant *χ*^2-^test may indicate a better fit.^27,30^ Multi-group CFA was performed to assess the measurement invariance of the HRSA across gender. Three levels of factorial invariance were measured: configural invariance (that the same factor structure exists across different groups), metric invariance (the factor loadings for the items are equal across different groups) and scalar invariance (both the factor loadings and the intercepts of the items are equal across different groups). Factorial invariance was established if ⊿CFI was less than 0.01 and ⊿RMSEA was less than 0.015.^31^ Factorial invariance was conducted with JASP 0.0.17.0.0 with a DWLS estimator, estimation for mean, and intercepts.

The polytomous rating scale model was used because all 14 items of the HRSA are Likert scales with scores ranging from 0 to 4.^32^ Parametric IRT analysis properties such as item difficulty, an information-weighted fit statistic (infit) mean square (MnSq) and outlier-sensitive fit statistic (outfit) MnSq, and thresholds (*τ*i1, *τ*i2, *τ*i3 and *τ*i4) were determined with the eRm R package in the snowIRT program of the JAMOVI 2.3.18, using marginal maximum likelihood estimates.^17^ Furthermore, Wright map person–item distribution and ICCs were used to show the graphical representation of the estimated item parameters.^17^ The differential item function (DIF) test was performed with the difNLR package of R in JAMOVI 2.3.18. DIF was estimated using these methods: model-adjacent,^33^ type of DIF (uniform and non-uniform),^33^ matching criterion z-score^34^ and Bonferroni multiple comparison adjustments. Item purification was not used as there were no concerns about DIF, and the HRSA tool is short. The difNLR:difORD function was used for ordinal data, which employs generalised logistic regression models for DIF estimation.^34^

Mean and s.d. of the HRSA scores, percentage distribution across item scores, Cronbach’s *α*-test, McDonald’s *ω*, item-rest correlations, skewness and kurtosis were determined by JAMOVI 2.3.18. Concurrent validity was evaluated by a receiver operating characteristic (ROC) curve analysis, using SPSS 23.0. A dichotomised score of the anxiety subscale of the DASS-21 was used as the state variable, and the HRSA total score as the test variable in the ROC curve analysis. A score of 10 or above on the anxiety subscale of the DASS-21 was taken to indicate moderate to severe anxiety.^25,35^ Further, the area under the curve analysis was performed. A cut-off score for the test measure HRSA total score was determined at the point of the highest value of accuracy, the score at which the sum of sensitivity and specificity is highest.

## Results

### Participants’ characteristics

The average value of age, grade point average (scale of 4.0), anxiety subscale score of the DASS-21 and the HRSA total score were 21.44 ± 2.30 years, 13.1 ± 0.58, 13.01 ± 9.14 and 15.02 ± 9.66, respectively (Table 1). Amhara and Oromo linguistic ethnicities formed the majority of the participants (63.5%). More than two-thirds of the participating students were male (67.8%). The prevalence of self-reported chronic disease/health conditions was high (18.9%). The presence of backlogs in previous examinations was common, with one in seven students reporting it (14.1%).

**Table 1:** Participant characteristics of the university students

Characteristics	Mean ± s.d./ frequency, *n* (%)
Age (years)	21.44 ± 2.30
Ethnicity	
Bench	6 (1.6)
Keffa	14 (3.8)
Amhara	168 (45.4)
Oromo	67 (18.1)
Tigray	4 (1.1)
Others	72 (19.5)
Did not report	39 (10.5)
Gender	
Male	251 (67.8)
Female	119 (32.2)
Grade (average GPA on scale of 0 to 4)	3.1 ± 0.58
Self-reported presence of chronic disease/conditions	
Yes	70 (18.9)
No	300 (81.1)
Backlog in the previous exams	
Yes	52 (14.1)
No	306 (82.7)
Did not report	12 (3.2)
Attendance in the classes	
Up to 50%	6 (1.6)
51–90%	38 (10.3)
91–100%	286 (77.3)
Did not report	40 (10.8)
Anxiety subscale DASS-21	13.01 ± 9.14
HRSA total score	15.02 ± 9.66

GPA, grade point average; DASS-21, Depression, Anxiety and Stress Scale - 21 Item; HRSA, Hamilton Rating Scale for Anxiety.

### Factor analysis

#### CFA

All three models performed very similarly, as indicated by multiple fit indices: CFI, GFI and IFI were above 0.95, and SRMR and RMSEA were < 0.06 for all three models (Table 2). Both two-factor models had slightly better values for fit indices in the CFA except, the parsimony-adjusted PNFI (Table 2), but had high interitem correlations of 0.84 and 0.87 (Supplementary Fig. 1). Therefore, the one-factor solution was deemed suitable. According to the criteria of Comrey and Lee, an average factor loading of 0.65 indicate a very good level of overlap in the variance of HRSA item scores (Table 3).^36^

**Table 2:** Fit statistics of the Hamilton Rating Scale for Anxiety scores in university students

Models	GFI	IFI	CFI	SRMR	RMSEA	*χ*^2^	d.f.	*P*-value
One-factor	0.989	0.989	0.989	0.058	0.054 (0.042–0.065)	158.771	77	<0.001
Two-factor (Rodriguez-Seijas et al 2020)^29^	0.991	0.992	0.992	0.053	0.046 (0.033–0.058)	134.707	76	<0.001
Two-factor (Hallit et al 2020)^24^	0.992	0.995	0.995	0.049	0.036 (0.021–0.050)	113.161	76	0.004

GFI, goodness of fit index; IFI, Bollen’s incremental fit index; CFI, comparative fit index; SRMR, standardised root mean square residual; RMSEA, root mean square error of approximation.

**Table 3:** Factor loading of some reported models of the Hamilton Rating Scale for Anxiety (HRSA) in university students

HRSA Item	Factor loadings									
	One-factor model		Two-factor model (Rodriguez-Seijas et al 2020)^29^				Two-factor model (Hallit et al 2020)^24^			
	Estimate	s.e.	Estimate	s.e.	Estimate	s.e.	Estimate	s.e.	Estimate	s.e.
HRSA_1	0.650	0.035	0.697	0.035			0.698	0.035		
HRSA_2	0.564	0.041	0.603	0.042			0.606	0.043		
HRSA_3	0.537	0.039			0.544	0.040	0.572	0.039		
HRSA_4	0.583	0.037	0.622	0.039			0.625	0.038		
HRSA_5	0.587	0.036	0.625	0.039			0.628	0.039		
HRSA_6	0.628	0.036	0.671	0.036			0.675	0.037		
HRSA_7	0.727	0.029			0.737	0.029			0.743	0.030
HRSA_8	0.703	0.033			0.711	0.033			0.716	0.033
HRSA_9	0.745	0.027			0.754	0.027			0.759	0.027
HRSA_10	0.735	0.029			0.743	0.029			0.748	0.029
HRSA_11	0.630	0.035			0.637	0.035			0.642	0.035
HRSA_12	0.686	0.033			0.694	0.033			0.700	0.033
HRSA_13	0.686	0.031			0.693	0.031			0.698	0.031
HRSA_14	0.615	0.036			0.622	0.036			0.628	0.036

#### Structural invariance: one-factor model multi-group CFA across gender groups

The configural model tests the basic structure of the latent variable across genders, the metric model tests the factor loadings of the latent variable to be equal across genders and the scalar model tests both the factor loadings and the intercepts of the latent variable to be equal across genders.

The *∆χ*^2^, *∆*CFI and *∆*SRMR values show the change in fit statistics between the current model and the previous model. If the *∆χ*^2^ value is significant at the 0.05 level, it indicates that the current model has a significantly worse fit than the previous model. Conversely, if the *∆χ*^2^ value is not significant, it suggests that the current model is not significantly worse than the previous model. The CFI and SRMR values should be close to 1 and 0, respectively, indicating a good fit. The RMSEA value should be less than 0.08 for an acceptable fit, and less than 0.05 for a good fit.

The results suggest that the configural model has a good fit, as the CFI and RMSEA values are both acceptable (Table 4). The metric and scalar models both have a slightly worse fit than the configural model, but they still have acceptable fit indices. The *∆χ*^2^ values between the configural and metric models and the metric and scalar models are not significant, indicating that there is no significant difference in fit between these models (Table 4). However, the *∆*RMSEA and *∆*SRMR values suggest that there may be some improvement in fit from the metric to the scalar model. In conclusion, measurement invariance is confirmed across gender subgroups (Table 4).

**Table 4:** Structural invariance of the one-factor model of the Hamilton Rating Scale for Anxiety in university students across gender groups

Model and comparisons	Fit statistics							
	*χ*^2^ (d.f.)	*∆χ*^2^ (*∆*d.f.)	CFI	*∆*CFI	SRMR	*∆*SRMR	RMSEA	*∆*RMSEA
M1: Configural	226.038 (154)		0.991		0.068		0.051	
M2: Metric	253.460 (167)		0.989		0.072		0.054	
M3: Scalar	277.205 (208)		0.991		0.070		0.043	
M2−M1		27.422 (13)		−0.002		0.004		0.003
M3−M2		23.745 (41)		0.002		−0.002		−0.011

CFI, comparative fit index; SRMR, standardised root mean square residual; RMSEA, root mean square error of approximation.

#### HRSA item analysis: classical theory parameters

There was no trend in the missing values of the HRSA scores: 0.4% missing values (19 values out of 5180) in 3.5% cases (13 out of 370) for eight HRSA item scores. There was no major issue of non-normality in HRSA item score distribution as the absolute value of skewness (highest value was 1.22 for HRSA item 10) and kurtosis (highest value was 0.68 for HRSA item 10) were less than 2 and 7, respectively (Table 5),^37^ as well as on the visual inspection. All HRSA items showed floor effect (>15% of respondents recorded the lowest possible score).^38,39^ No ceiling/floor effect was seen for the HRSA total score (range: 0–52; 3.5% (13 students) recorded the lowest score of 0, and none recorded the highest score of 56).^38,39^

**Table 5:** Item analysis, internal homogeneity, of Hamilton Rating Scale for Anxiety (HRSA) scores in university students

Items of the HRSA	Mean	s.d.	If item deleted		Correlation		Skewness			Kurtosis			Percentage distribution across item scores					
			Cronbach’s *α*	McDonald’s *ω*	Item total	Item rest	Statistic	s.e.	*z*	Statistic	s.e.	*z*	0	1	2	3	4	Missing values
HRSA_1	0.99	0.96	0.87	0.87	0.62*	0.60*	0.77	0.13	6.08	0.24	0.25	0.96	36.8	33.5	23.5	3.5	1.9	0.8
HRSA_2	1.29	1.11	0.87	0.88	0.59*	0.47*	0.50	0.13	3.94	−0.48	0.25	−1.88	30.0	28.4	28.6	9.5	3.5	0.0
HRSA_3	1.37	1.17	0.87	0.88	0.55*	0.47*	0.53	0.13	4.17	−0.55	0.25	−2.17	27.6	30.3	24.9	11.9	5.4	0.0
HRSA_4	1.22	1.16	0.87	0.87	0.59*	0.50*	0.76	0.13	5.95	−0.23	0.25	−0.92	32.7	31.6	20.0	9.2	5.4	1.1
HRSA_5	1.18	1.20	0.87	0.87	0.60*	0.50*	0.80	0.13	6.33	−0.28	0.25	−1.10	36.8	29.2	18.6	9.5	5.9	0.0
HRSA_6	1.30	1.20	0.87	0.87	0.63*	0.55*	0.63	0.13	4.95	−0.50	0.25	−1.97	31.1	29.7	22.2	11.1	5.9	0.0
HRSA_7	1.07	1.09	0.86	0.87	0.71*	0.65*	0.70	0.13	5.48	−0.48	0.25	−1.89	40.3	26.5	21.4	9.7	1.9	0.3
HRSA_8	0.90	1.04	0.87	0.87	0.66*	0.59*	0.99	0.13	7.82	0.20	0.25	0.79	45.7	28.1	16.5	7.3	2.2	0.3
HRSA_9	0.93	1.10	0.87	0.87	0.67*	0.61*	1.05	0.13	8.18	0.25	0.25	0.99	46.2	26.5	15.4	7.6	3.2	1.1
HRSA_10	0.83	1.07	0.87	0.87	0.67*	0.59*	1.22	0.13	9.62	0.68	0.25	2.69	51.6	25.7	12.7	7.0	2.7	0.3
HRSA_11	1.06	1.16	0.87	0.87	0.61*	0.50*	0.89	0.13	6.93	−0.12	0.25	−0.48	43.0	24.3	19.5	7.8	4.3	1.1
HRSA_12	0.86	1.04	0.87	0.87	0.64*	0.56*	1.14	0.13	8.97	0.62	0.25	2.45	48.6	27.0	15.7	5.7	2.7	0.3
HRSA_13	0.90	1.08	0.87	0.87	0.64*	0.58*	1.10	0.13	8.65	0.49	0.25	1.92	48.6	25.1	17.6	5.4	3.2	0.0
HRSA_14	1.11	1.12	0.87	0.87	0.60*	0.51*	0.86	0.13	6.75	0.08	0.25	0.32	37.6	29.2	22.7	5.7	4.9	0.0

*P < 0.001.

#### HRSA item analysis: Rasch rating scale model parameters

HRSA item 10 (respiratory symptoms) was the most difficult item, and HRSA item 3 (fears of the dark, strangers, etc.) was the easiest task, as indicated by item difficulty/severity scores of 0.333 and −0.380, respectively (Table 6). Infit and outfit statistics of the HRSA item scores were in the desirable range: 0.6–1.4. Four threshold estimates (*τ*i1, *τ*i2, *τ*i3 and *τ*i4) were determined for all 14 HRSA items, as these are scored from 0 to 4 with five response levels. For all 14 HRSA item scores, the thresholds were ordered (*τ*i1 < *τ*i2 < *τ*i3 < *τ*i4) (Table 6).

**Table 6:** Summary of item difficulty, polytomous mean-square fit statistics (infit, outfit) and threshold (*τ*i) statistics of the rating scale model: Hamilton Rating Scale for Anxiety (HRSA)

Items of the HRSA	Severity	s.e.	Outfit MnSq	Infit MnSq	Threshold 1 (*τ*i1)	Threshold 2 (*τ*i2)	Threshold 3 (*τ*i3)	Threshold 4 (*τ*i4)
HRSA_1	0.096	0.062	0.782	0.935	0.089	0.658	1.560	1.650
HRSA_2	−0.284	0.058	1.022	1.124	−0.264	0.304	1.200	1.300
HRSA_3	−0.380	0.057	1.113	1.146	−0.353	0.215	1.120	1.210
HRSA_4	−0.202	0.059	1.094	1.103	−0.189	0.380	1.280	1.370
HRSA_5	−0.147	0.059	1.179	1.113	−0.137	0.432	1.330	1.430
HRSA_6	−0.297	0.058	1.023	1.058	−0.277	0.292	1.190	1.290
HRSA_7	−0.003	0.061	0.784	0.792	−0.003	0.566	1.470	1.560
HRSA_8	0.228	0.064	0.915	0.976	0.212	0.781	1.680	1.780
HRSA_9	0.183	0.064	0.958	0.826	0.170	0.739	1.640	1.730
HRSA_10	0.333	0.066	1.020	0.893	0.311	0.879	1.780	1.870
HRSA_11	0.005	0.061	1.177	1.115	0.004	0.573	1.470	1.570
HRSA_12	0.295	0.065	0.992	0.917	0.275	0.843	1.740	1.840
HRSA_13	0.232	0.064	0.985	0.868	0.216	0.784	1.680	1.780
HRSA_14	−0.058	0.060	1.042	1.137	−0.054	0.515	1.420	1.510

**Fig. 1:**
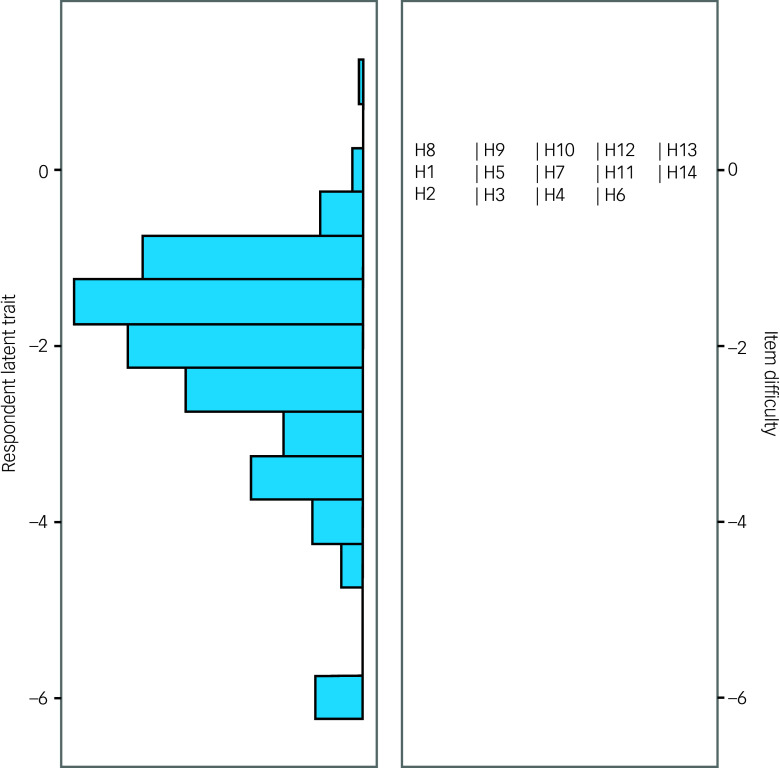
Wright map person–item distribution for individual items of the Hamilton Rating Scale for Anxiety (HRSA). H1 to H14 are items of the HRSA.

All of the HRSA items showed invariance across gender groups indicated by non-significant likelihood ratio chi-squared statistics at adjusted *P*-values for both uniform and non-uniform estimates (Table 7). A visual inspection of the Wright map (Fig. 1) shows that the width of the spread of the person’s ability shown on the left panel, and the item difficulty level on the right panel do not match. The item difficulty level of the HRSA items do mostly correspond with the people with higher ability levels. An inspection of ICCs revealed that the response level functioned as expected for all of the HRSA item scores. For instance, at a latent dimension of 1.0 (Supplementary Fig. 2), the probability of getting a third response level (approximately 38%) is nearly similar for all HRSA item scores.

**Table 7:** Differential item function (DIF) test on the Hamilton Rating Scale for Anxiety (HRSA) in university students across gender groups

Items of the HRSA tool	Uniform DIF estimate			Non-uniform DIF estimate		
	Likelihood ratio chi-squared statistics	Unadjusted *P*-value	Adjusted *P*-value	Likelihood ratio chi-squared statistics	Unadjusted *P*-value	Adjusted *P*-value
HRSA_1	0.442	0.506	1.00	0.711	0.399	1.00
HRSA_2	1.023	0.312	1.00	0.580	0.447	1.00
HRSA_3	0.178	0.673	1.00	0.882	0.348	1.00
HRSA_4	1.039	0.308	1.00	2.205	0.138	1.00
HRSA_5	4.511	0.034	0.47	0.137	0.712	1.00
HRSA_6	0.300	0.584	1.00	0.011	0.918	1.00
HRSA_7	0.014	0.907	1.00	0.441	0.507	1.00
HRSA_8	1.327	0.249	1.00	0.315	0.574	1.00
HRSA_9	0.185	0.667	1.00	1.041	0.308	1.00
HRSA_10	0.358	0.550	1.00	1.926	0.165	1.00
HRSA_11	0.607	0.436	1.00	0.277	0.599	1.00
HRSA_12	1.285	0.257	1.00	0.084	0.772	1.00
HRSA_13	0.938	0.333	1.00	0.210	0.646	1.00
HRSA_14	6.497	0.011	0.15	0.212	0.645	1.00

#### Internal consistency and item discrimination

McDonald’s *ω* and Cronbach’s *α* for the HRSA tool were 0.88, and 0.88, respectively. McDonald’s *ω* if the item was deleted, and Cronbach’s *α* if the item was deleted ranged from 0.88 to 0.87, and 0.87 to 0.86, respectively (Table 5). The correlation coefficients of the item-total and item-rest scores of the HRSA ranged from 0.71 to 0.55, and 0.65 to 0.47, respectively.

#### Convergent validity: correlation between HRSA score and self-reported measure of anxiety

All of the correlation coefficients were between HRSA scores (item and total), and the anxiety subscale of the DASS-21 was significant and varied between 0.29 and 0.56 (Supplementary Table 2). The ROC test showed that an HRSA total score of 13.5 and above had a sensitivity of 72.1% and specificity of 74.4%, respectively, to screen cases of moderate-severe level of anxiety as determined by the score of the anxiety subscale of the DASS-21. The AUC was 0.78 (95% CI 0.73–0.83, *P* < 0.001) (Supplementary Fig. 3).

## Discussion

To the best of our knowledge, this is the first study to employ an appropriate Rasch rating scale approach for IRT-based psychometric measures, including the ICC and Wright map; as well as the first to record statistical evidence for both structural- and item-level measurement invariance, for the one-factor structure of the HRSA across gender groups. It is also the first comprehensive psychometric study of the Hamilton Rating Scale for Anxiety on a sample of collegiate young adults in an African country, and one of very few studies on the factor analysis of the HRSA that verified the assumptions of various sample size adequacy measures, including the KMO test, Bartlett’s test of sphericity and inter-item correlation matrix. In brief, the study found evidence that HRSA showed very good psychometric properties (summary in Table 8) in this sample of university students in Ethiopia.

**Table 8:** Summary of psychometric tests performed in the university students

Name of the psychometric test/index	Outcome/inference	Pass/fail/not applicable
CFA		
Fit indices	No favoured model out of three, although parsimony-adjusted fit index favours a one-factor model	Inconclusive
Interfactor correlation coefficient	One-factor model favoured because of high interfactor correlation coefficient (*r* = 0.84 and 0.87)	Pass for one-factor model
Multi-group CFA across genders	Configural model has a good fit, metric and scalar models have acceptable fit indices	Pass for one-factor model
HRSA item analysis: classical theory parameters		
Missing values	No trend in missing values	Pass
Distribution (skewness, kurtosis, visual inspection)	No major issue of non-normality	Pass
Ceiling/floor effect	No ceiling/floor effect for HRSA total score, but floor effect in HRSA item scores	Pass for HRSA total score
HRSA item analysis: Rasch rating scale model parameters		
Infit and outfit statistics	In the desirable range	Pass
Threshold estimates	Ordered as necessary	Pass
Item difficulty level	Items assessing somatic symptoms had higher difficulty levels, whereas those assessing psychic anxiety had lower difficulty levels	Not applicable
Wright map	Spread of the person’s ability versus the item difficulty level was asymmetrical	Fail
Item characteristic curves	Although slopes were ordered, there was almost overlap in category 3 and category 4 response slopes	Partial pass, response category 3 and 4 may be fused
DIF	All HRSA items and total scores had non-significant difference in DIF across gender	Pass
Internal consistency and item discrimination		
McDonald’s *ω* and Cronbach’s *α* McDonald’s *ω* if the item was deleted, and Cronbach’s *α* if the item was deleted	All above 0.86, so scale has very good internal consistency	Pass
Item-total and the item-rest score correlation coefficient	Moderate to strong correlation coefficient, so scale has good internal homogeneity	Pass
Convergent validity: correlation between HRSA score and self-reported measure of anxiety		
Correlation coefficients were between HRSA scores (item and total) and the anxiety subscale of the DASS-21	HRSA item and total scores significantly correlated with anxiety subscale of DASS-21	Pass
ROC curve analysis	Adequate level of sensitivity, specificity and area under the curve for HRSA total score with respect to DASS-21 anxiety subscale score	Pass

CFA, confirmatory factor analysis; HRSA, Hamilton Rating Scale for Anxiety; DIF, differential item function; DASS-21, Depression, Anxiety and Stress Scale 21 Items; ROC, receiver operating characteristic.

### CFA and structural invariance

A one-factor structure of the HRSA was deemed suitable because the comparative CFA was inconclusive, as revealed by similar fit indices for the three models. However, high interfactor correlation coefficients for the two two-factor models made them untenable because of the concerns of divergent validity of the factor constructs. High interfactor correlation coefficients (0.85 and above) violate divergent validity requirements for the different latent constructs.^27,40^ Moreover, the parsimony adjusted index of the PNFI was slightly higher for one-factor model. Similarly, a study conducted among 725 adults with or without symptoms of depression reported an acceptable one-factor model of HRSA that explained 34.6 and 38.5% of variance across groups.^29^ The study further concluded that the anxious-distress specifier evaluates a unidimensional construct with close associations to both the psychological and somatic manifestations of anxiety.^29^ This indicates that HRSA measures both physical and psychological manifestations as a unidimensional construct. It is difficult to establish invariance, but given the outcome of the multi-group CFA across genders in this study, it is unlikely that responses vary by gender. This further supports the unidimensionality of the HRSA.^40^ The one-factor structure was found to show invariance at four levels of measurements, i.e. configural, metric, scalar and strict across gender groups. Few studies reported structural invariance across gender for HRSA, although it has been in wide clinical and research use since its development some six decades ago. There is much disparity in the findings about the structural validity of the HRSA, with studies reporting validity of one-, two- and three-factor models in different demographics such as adolescents, non-clinical samples, people living with Parkinson’s disease and patients visiting psychiatry clinics.^15,16,23,29^ The disparity in findings of previous studies on the dimensionality of the HRSA may be partly explained by the non-reporting of structural invariance measures.^15,16,23,29^

For a questionnaire to be measurement invariant, it must measure identical constructs with the same structure across all groups. Using a multi-group CFA, our procedure for testing the measurement invariance of the 14-item questionnaire included three sequential steps requiring increasingly stringent equality constraints on between-group model parameters. This is the first study to demonstrate measurement invariance for the one-factor solution of the HRSA across three levels, i.e. configural, metric and scalar, across gender groups. The results of these tests provided additional support for the one-factor model of the HRSA in the study population.

### Item evaluation with classical theory

Results indicated no significant deviation from the univariate distribution for items, factors and total scores of the HRSA; this suggests that the score distribution followed a pattern that is typical for a general population.^21^ This lends credibility to the study’s findings as a whole. In addition, the absence of a ceiling/floor effect for the HRSA total score suggests that even at extreme scores, HRSA total score can differentiate between groups. Although all of the HRSA item scores had floor effects, this may be attributable to the research population’s non-clinical makeup – young people who attend universities. Similarly, a clinical construct for assessing insomnia severity showed floor effects for item scores, but not for the construct level measures.^37^

### Convergent validity: correlation between HRSA score and self-reported measure of anxiety

A significant positive correlation with the self-reported measure of anxiety indicated the convergent validity of the HRSA scores. However, all of the correlations between HRSA item scores and the anxiety subscale of the DASS-21 were weak, and the correlation between the HRSA total score and the anxiety subscale of the DASS-21 was moderate and positive.^25^ Similarly, HRSA scores were shown to have low-moderate correlations with self-reported measures of anxiety and fear among American adolescents.^25^ Furthermore, in people living with Parkinson’s disease, a modest level of convergent validity was found for the HRSA score.^15^ Our results concerning convergent validity are similar to previous studies,^23,15^ which may suggest a further modification in scale items to improve convergent validity. However, it may be important to highlight two important issues that might explain the reasons for this modest correlation. First, HRSA comprises items that assess anxiety symptoms across many physiological systems, unlike other measures that usually consider only psychological symptoms. Second, HRSA is an expert-administered tool, whereas the DASS-21 measures used for convergent validity assessment by previous studies are mostly self-reported measures. Tool item score assignment is done by experts.^13,15,17,23^ Therefore, it is pragmatic to expect that some of the variances may not be accounted for when correlating an expert-level evaluation with self-reported measures. Future studies may better explore the convergent and divergent validity of HRSA by using similar measures administered by experts/health professionals. Furthermore, the convergent validity of the HRSA with respect to the anxiety subscale of DASS-21 was evidenced by the adequate level of sensitivity, specificity and AUC.

### HRSA item analysis: Rasch rating scale model parameters

It would be pragmatic to mention here that a direct comparison with previous studies is not possible because, to the best of our knowledge, there are no reports of item difficulty level or MnSq infit/outfit of HRSA scores based on the rating scale model. The item difficulty analysis in this study showed that the easier items were related to items 3 (fears), 6 (depressed mood), 2 (tension) and 4 (insomnia), whereas the more challenging items were related to items 10 (respiratory symptoms), 12 (genitourinary symptoms), 13 (autonomic symptoms) and 8 (somatic sensory symptoms). Therefore, there appears to be a trend wherein items with higher difficulty levels were taking appraisal of somatic symptoms, whereas items assessing psychic anxiety had lower difficulty levels.^13^ Forjaz et al found that the Rasch analysis of HRSA in Parkinson’s disease showed that the three most difficult items were item 9 (cardiovascular symptoms), item 14 (behaviour at interview) and item 10 (respiratory symptoms); easier items were item 1 (anxious mood), item 8 (somatic sensory) and item 2 (tension).^17^ As evident, both studies have similar findings for item 10 (respiratory symptoms) and item 2 (tension). However, most of the trends regarding difficulty level were not similar in both studies.^17^ This may be related to the differences in the statistical approach and sample characteristics: Forjaz et al used Rasch analysis in patients with Parkinson’s disease, whereas we implemented a Rating scale model in university-attending young adults^17^ who were enrolled in health science courses. The findings of this study do support using the HRSA tool practically in university students in Ethiopia. Future research may further explore the culture specific development and adaptation, and possibly help establish an evidence-based clinical application in the Ethiopian population in general.

The outfit statistic is sensitive to unexpected observations by person or item, whereas the infit statistic is sensitive to unexpected patterns where residuals are close to estimated individual abilities.^17^ In general, MnSq fit indices in the range of 0.5–1.5 imply that the measurement done by item scores is productive.^17^ Greater values indicate underfit between the items and the model, whereas lower values indicate overfit. An MnSq of 0.6–1.4 represents the optimal range for rating scale surveys.^17^ In this study, the range was 0.78–1.18, indicating that HRSA items are not ideal for rating scale surveys or clinical observation.^17^ Most of the HRSA items are overloaded and sometimes take an appraisal of as many as 11 signs and symptoms, as in the case of item 11 (gastrointestinal symptoms). Therefore, our findings suggest that clinical implementation of the HRSA, even by experts, may benefit from attempts to simplify the HRSA items.

The response-level thresholds were ordered, but the difference between *τ*i3, and *τ*i4 were narrow for all items. This was also evident in the near overlap in category 3 and category 4 response slopes in the ICCs. The statistical consideration from these two findings, i.e. a narrowed gap in *τ*i3 and *τ*i4, and near overlap in category 3 and category 4 response slopes, suggest that category 3 and category 4 responses may be fused. However, a degree of caution may be suggested because HRSA items are highly loaded, and a further decrease in the number of response categories may result in ambiguities in response allocation by the interviewers.

A visual inspection of the Wright map (Fig. 1) shows that the spread of the person’s ability shown on the left panel and the item difficulty level on the right panel were asymmetrical. The item difficulty levels of the HRSA items mostly correspond with persons with higher ability levels. The results are in alignment with the expert interviewer-administered nature of the HRSA. This implies that more efforts are needed to possibly simplify and decrease the difficulty levels of some of the HRSA items. This may help attain a comparative width of ability distribution with the item difficulty levels.^17^ Finally, in the present study, item-level invariance was noted for all the HRSA items across gender groups. Forjaz et al found evidence for item-level invariance for only two items of the HRSA Rasch analysis in Parkinson’s disease.^17^

### Limitations

It is important to highlight some limitations that may aid comprehension of the generalisation and implications of the findings. The results may not be generalisable to the general population because the student population differs from those of the general population. The study sample had more male students in a close age range, mostly from the health sciences discipline; some of these courses are more challenging. Therefore, a wider generalisability may need further exploration with more representative samples. To maximise participation, convenient sampling was used; however, such a sampling procedure may theoretically limit generalisability. However, it is appropriate to note that the study’s sample size was substantial. In addition, the absence of significant skewness/kurtosis issues reinforces the representativeness of the study sample. Moreover, robust measures of psychometric validity testing using both classical theory and Rasch rating theory parameters were utilised in this study. Additionally, future research may investigate temporal measurement invariance, as well as invariance across other sociodemographic parameters.

In conclusion, classical and IRT analysis measures demonstrated that the HRSA possessed robust psychometric validity. Nonetheless, there is evidence that future research efforts could increase the practical use of the HRSA. Such efforts may investigate (a) simplifying HRSA items, (b) exploring response levels further, (c) establishing item-level invariance across different demographic characteristics and (d) attempting to increase symmetry in a person’s ability and item difficulty levels.

## Supporting information

Manzar et al. supplementary material 1 Manzar et al. supplementary material

Manzar et al. supplementary material 2 Manzar et al. supplementary material

Manzar et al. supplementary material 3 Manzar et al. supplementary material

Manzar et al. supplementary material 4 Manzar et al. supplementary material

Manzar et al. supplementary material 5 Manzar et al. supplementary material

## Data Availability

The raw data supporting the conclusions of this article are submitted as Supplementary Material.

## Supplementary material

The supplementary material is available online at https://doi.org/10.1192/bjo.2025.10055
