# Supplementary material for: Hamilton Rating Scale for Anxiety: exploring validity with robust measures of classical theory parameters and a rating scale model in university students
Source: BJPsych Open. 2025 Aug 12;11(5):e176. doi: 10.1192/bjo.2025.10055 (PMC12451730; doi:10.1192/bjo.2025.10055)
Supplement: Manzar et al. supplementary material 1 — Manzar et al. supplementary material [file S2056472425100550sup001.docx]

Supplement Figure 1. Factor structures of the of the Hamilton Anxiety Rating Scale among the university students


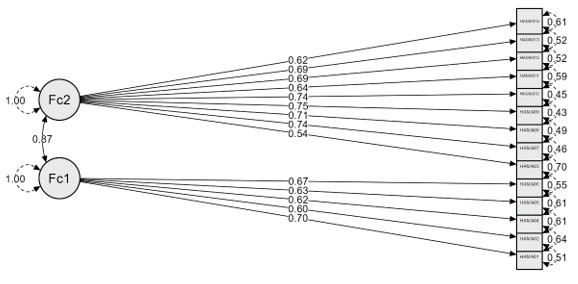


A 2-Factor model (Rodriguez-Seijas et al 2020) of the Hamilton Anxiety Rating Scale among the university students


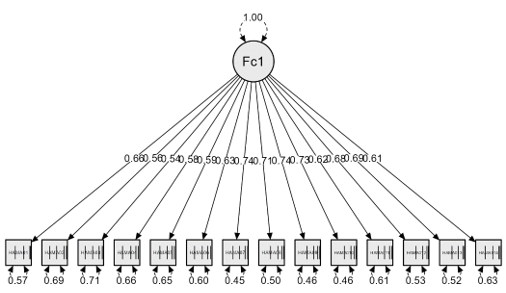


A 1-Factor model of the Hamilton Anxiety Rating Scale among the university students


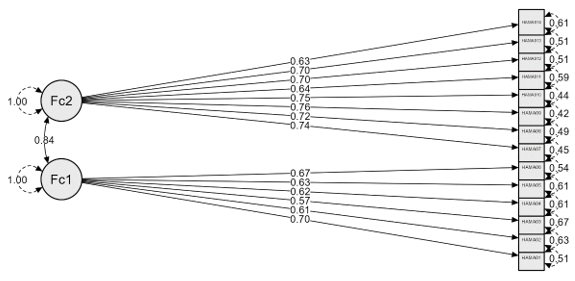


A 2-Factor model (Hallit et al 2020) of the Hamilton Anxiety Rating Scale among the university students

All coefficients are standardized. *Circles* latent variables, *rectangles* measured variables, error terms, *single-headed arrows* between *Circles* and *rectangles* factor loadings, *two-headed arrows on circles, and rectangles* error terms
