# Supplementary material for: Hamilton Rating Scale for Anxiety: exploring validity with robust measures of classical theory parameters and a rating scale model in university students
Source: BJPsych Open. 2025 Aug 12;11(5):e176. doi: 10.1192/bjo.2025.10055 (PMC12451730; doi:10.1192/bjo.2025.10055)
Supplement: Manzar et al. supplementary material 2 — Manzar et al. supplementary material [file S2056472425100550sup002.docx]

##
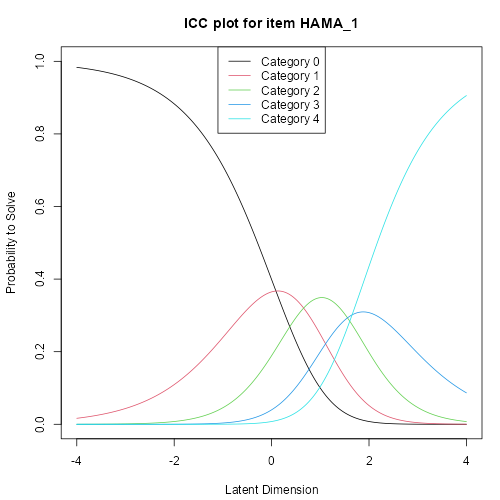

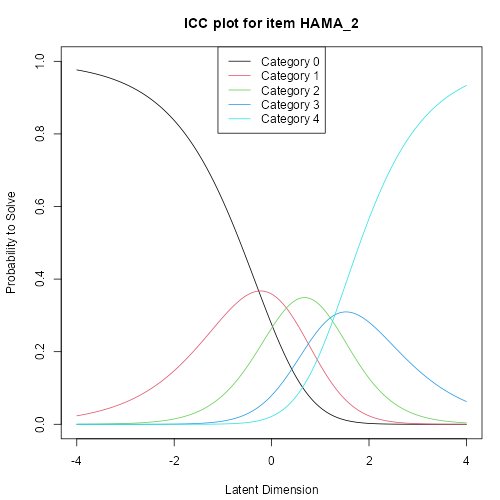

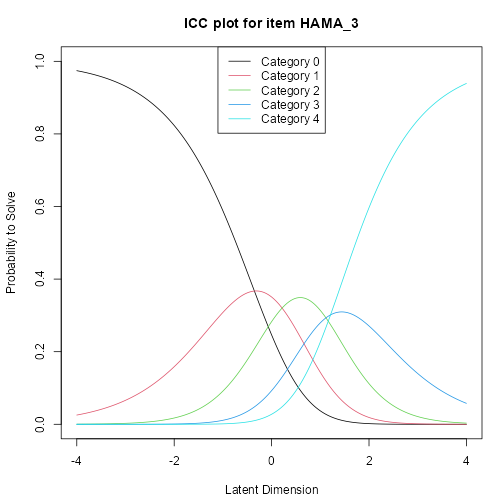

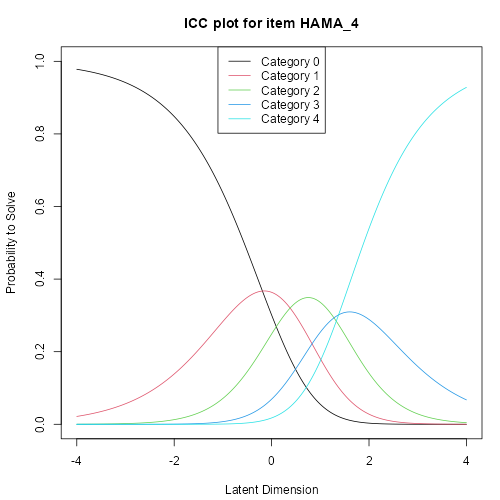

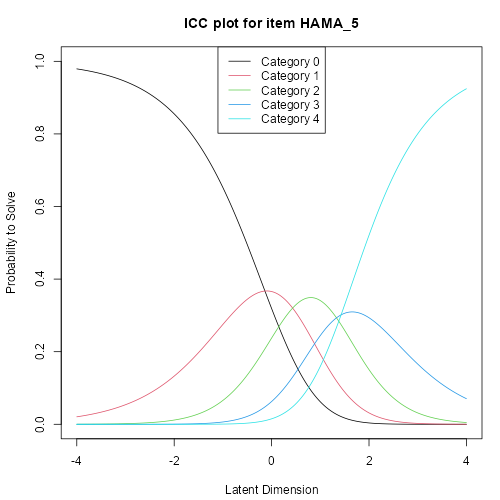

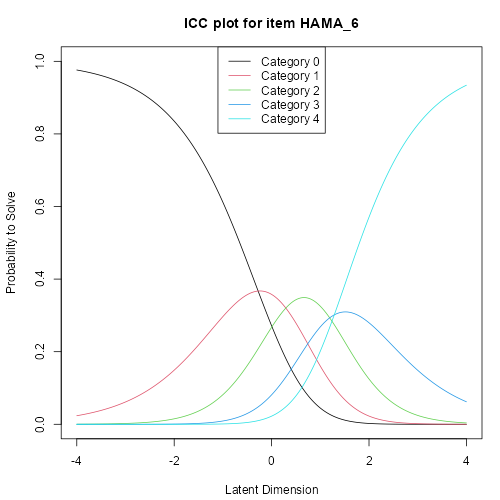


##
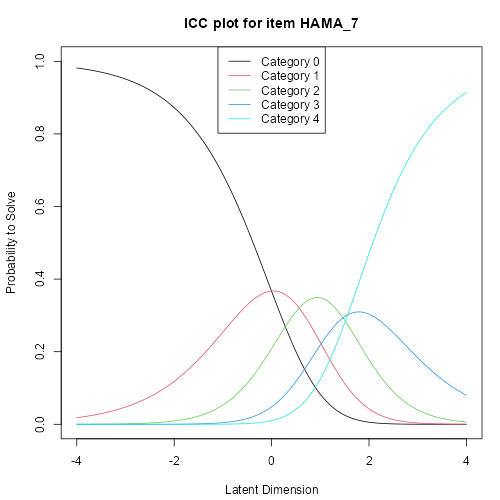

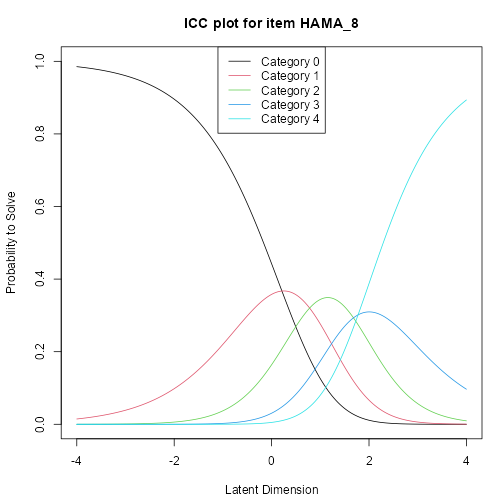

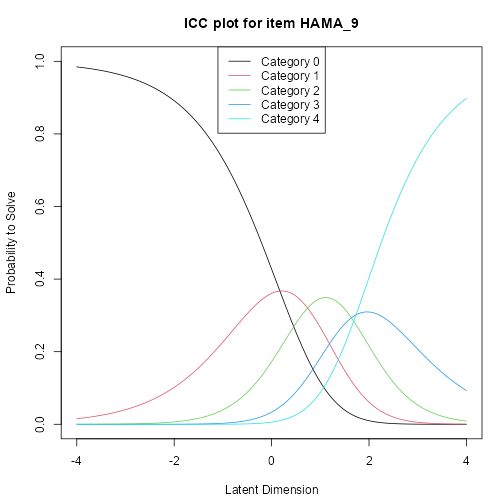

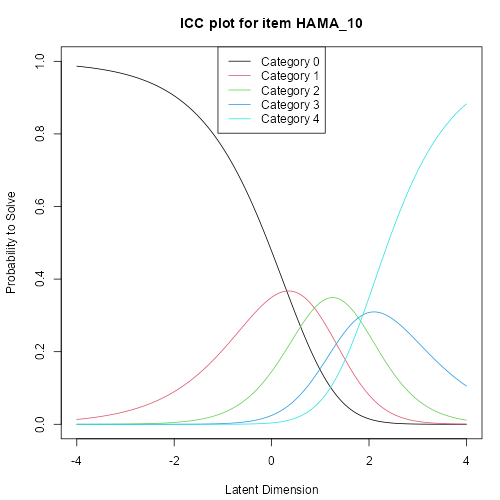

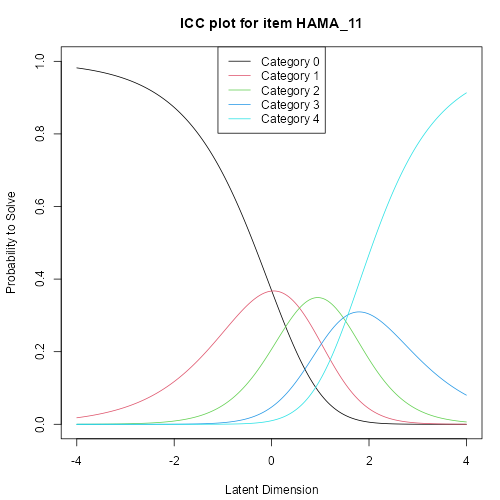

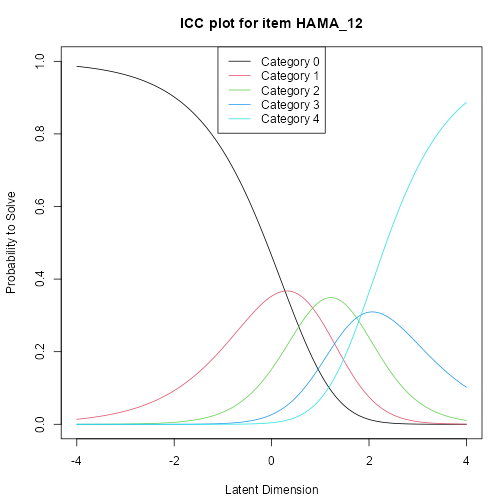


##
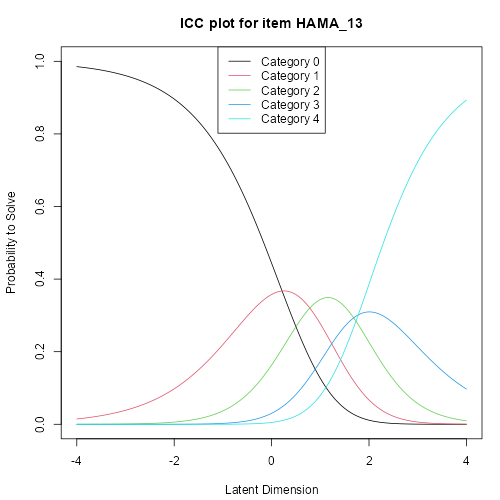

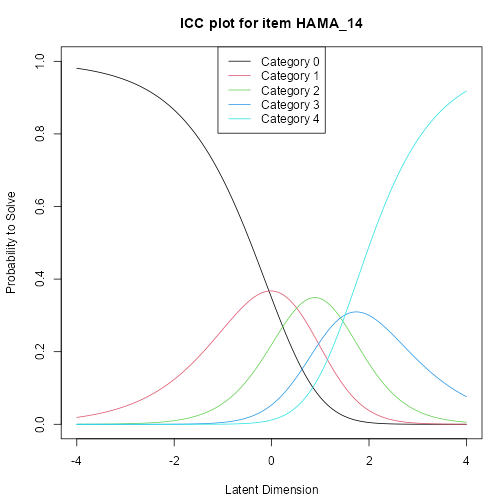


Supplement Figure 2. Item characteristic curves of the Hamilton anxiety rating scale (HAM-A) scores among university students
