# Supplementary material for: Hamilton Rating Scale for Anxiety: exploring validity with robust measures of classical theory parameters and a rating scale model in university students
Source: BJPsych Open. 2025 Aug 12;11(5):e176. doi: 10.1192/bjo.2025.10055 (PMC12451730; doi:10.1192/bjo.2025.10055)
Supplement: Manzar et al. supplementary material 3 — Manzar et al. supplementary material [file S2056472425100550sup003.docx]

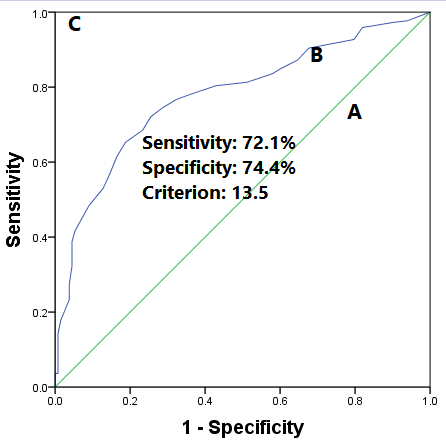


Supplement Figure 3 Receiver Operator curves (A) line of no discrimination with AUC of 0.5, (B) line of experimental test (HAM-A total score; 0.78, p < 0.001), and (C) line of perfect test with an AUC of 1.0 in university students.

A dichotomized score of the anxiety sub-scale of the DASS-21 was used as the state variable, and the HAM-A total score as the test variable in the ROC curve analysis. A score of 10 and above on the anxiety sub-scale of the DASS-21 was taken to indicate moderate to severe anxiety^27, 38^.

HAM-A: Hamilton Anxiety Rating Scale; DASS-21: Depression, Anxiety and Stress Scale - 21
