# Supplementary material for: Hamilton Rating Scale for Anxiety: exploring validity with robust measures of classical theory parameters and a rating scale model in university students
Source: BJPsych Open. 2025 Aug 12;11(5):e176. doi: 10.1192/bjo.2025.10055 (PMC12451730; doi:10.1192/bjo.2025.10055)
Supplement: Manzar et al. supplementary material 4 — Manzar et al. supplementary material [file S2056472425100550sup004.docx]

Supplement Table 1 Inter-item Correlation matrix (Spearman rho) of the Hamilton Anxiety Rating Scale (HAM-A) scores in Ethiopian university students

|  | HAMA_1 | HAMA_2 | HAMA_3 | HAMA_4 | HAMA_5 | HAMA_6 | HAMA_7 | HAMA_8 | HAMA_9 | HAMA_10 | HAMA_11 | HAMA_12 | HAMA_13 | HAMA_14 |
| --- | --- | --- | --- | --- | --- | --- | --- | --- | --- | --- | --- | --- | --- | --- |
| HAMA_1 | — |  |  |  |  |  |  |  |  |  |  |  |  |  |
| HAMA_2 | 0.47 | — |  |  |  |  |  |  |  |  |  |  |  |  |
| HAMA_3 | 0.34 | 0.30 | — |  |  |  |  |  |  |  |  |  |  |  |
| HAMA_4 | 0.32 | 0.36 | 0.39 | — |  |  |  |  |  |  |  |  |  |  |
| HAMA_5 | 0.34 | 0.32 | 0.30 | 0.26 | — |  |  |  |  |  |  |  |  |  |
| HAMA_6 | 0.40 | 0.38 | 0.34 | 0.36 | 0.33 | — |  |  |  |  |  |  |  |  |
| HAMA_7 | 0.43 | 0.32 | 0.38 | 0.45 | 0.41 | 0.42 | — |  |  |  |  |  |  |  |
| HAMA_8 | 0.35 | 0.33 | 0.28 | 0.32 | 0.35 | 0.35 | 0.47 | — |  |  |  |  |  |  |
| HAMA_9 | 0.36 | 0.25 | 0.26 | 0.29 | 0.38 | 0.32 | 0.41 | 0.52 | — |  |  |  |  |  |
| HAMA_10 | 0.35 | 0.39 | 0.29 | 0.27 | 0.33 | 0.35 | 0.47 | 0.40 | 0.56 | — |  |  |  |  |
| HAMA_11 | 0.23 | 0.31 | 0.34 | 0.28 | 0.28 | 0.31 | 0.33 | 0.41 | 0.44 | 0.49 | — |  |  |  |
| HAMA_12 | 0.33 | 0.21 | 0.27 | 0.34 | 0.40 | 0.29 | 0.39 | 0.46 | 0.47 | 0.42 | 0.47 | — |  |  |
| HAMA_13 | 0.33 | 0.30 | 0.24 | 0.35 | 0.30 | 0.35 | 0.40 | 0.47 | 0.39 | 0.44 | 0.38 | 0.40 | — |  |
| HAMA_14 | 0.35 | 0.29 | 0.22 | 0.33 | 0.31 | 0.37 | 0.36 | 0.33 | 0.43 | 0.38 | 0.30 | 0.36 | 0.45 | — |

All coefficients were significant, *p* < 0.01
