# Supplementary material for: Hamilton Rating Scale for Anxiety: exploring validity with robust measures of classical theory parameters and a rating scale model in university students
Source: BJPsych Open. 2025 Aug 12;11(5):e176. doi: 10.1192/bjo.2025.10055 (PMC12451730; doi:10.1192/bjo.2025.10055)
Supplement: Manzar et al. supplementary material 5 — Manzar et al. supplementary material [file S2056472425100550sup005.docx]

Supplement Table 2 Convergent validity: Spearman rho of the Hamilton Anxiety Rating Scale (HAM-A) scores with the anxiety sub-scale score of the Depression, anxiety, and stress scale-21 (DASS-21) in Ethiopian university students

| Items of the HAM-A | Correlation coefficient with the  Anxiety sub-scale score  of the DASS-21 |
| --- | --- |
| HAMA_1 | .402^*^ |
| HAMA_2 | .291^*^ |
| HAMA_3 | .296^*^ |
| HAMA_4 | .327^*^ |
| HAMA_5 | .320^*^ |
| HAMA_6 | .324^*^ |
| HAMA_7 | .414^*^ |
| HAMA_8 | .368^*^ |
| HAMA_9 | .399^*^ |
| HAMA_10 | .429^*^ |
| HAMA_11 | .303^*^ |
| HAMA_12 | .370^*^ |
| HAMA_13 | .360^*^ |
| HAMA_14 | .367^*^ |
| HAMA_total score | .556^*^ |

^*^*p<.01*
